# Modelling the impact of hybrid immunity on future COVID-19 epidemic waves

**DOI:** 10.1186/s12879-024-09282-4

**Published:** 2024-04-16

**Authors:** Thao P. Le, Isobel Abell, Eamon Conway, Patricia T. Campbell, Alexandra B. Hogan, Michael J. Lydeamore, Jodie McVernon, Ivo Mueller, Camelia R. Walker, Christopher M. Baker

**Affiliations:** 1 School of Mathematics and Statistics, The University of Melbourne, Grattan Street, Melbourne, 3010 Victoria Australia; 2 Melbourne Centre for Data Science, The University of Melbourne, Grattan Street, Melbourne, 3010 Victoria Australia; 3 Centre of Excellence for Biosecurity Risk Analysis, The University of Melbourne, Grattan Street, Melbourne, 3010 Victoria Australia; 4 Population Health & Immunity Division, Walter and Eliza Hall Institute of Medical Research, 1G Royal Parade, Melbourne, 3052 Victoria Australia; 5 Department of Infectious Diseases at the Peter Doherty Institute for Infection and Immunity, The University of Melbourne, 792 Elizabeth St, Melbourne, 3000 Victoria Australia; 6 Melbourne School of Population and Global Health, The University of Melbourne, Bouverie St, Carlton, 3053 Victoria Australia; 7 School of Population Health, University of New South Wales, Sydney, 2033 New South Wales Australia; 8 MRC Centre for Global Infectious Disease Analysis, Jameel Institute, School of Public Health, Imperial College London, Exhibition Road, London, SW7 2AZ United Kingdom; 9 Department of Econometrics and Business Statistics, Monash University, Wellington Road, Melbourne, 3800 Victoria Australia; 10 Victorian Infectious Diseases Reference Laboratory Epidemiology Unit, The Royal Melbourne Hospital at the Peter Doherty Institute for Infection and Immunity, 792 Elizabeth St, Melbourne, 3000 Victoria Australia; 11 Department of Medical Biology, The University of Melbourne, Grattan Street, Melbourne, 3010 Victoria Australia

**Keywords:** Epidemiology, Mathematical modelling, Vaccination, Variants

## Abstract

**Background:**

Since the emergence of SARS-CoV-2 (COVID-19), there have been multiple waves of infection and multiple rounds of vaccination rollouts. Both prior infection and vaccination can prevent future infection and reduce severity of outcomes, combining to form hybrid immunity against COVID-19 at the individual and population level. Here, we explore how different combinations of hybrid immunity affect the size and severity of near-future Omicron waves.

**Methods:**

To investigate the role of hybrid immunity, we use an agent-based model of COVID-19 transmission with waning immunity to simulate outbreaks in populations with varied past attack rates and past vaccine coverages, basing the demographics and past histories on the World Health Organization Western Pacific Region.

**Results:**

We find that if the past infection immunity is high but vaccination levels are low, then the secondary outbreak with the same variant can occur within a few months after the first outbreak; meanwhile, high vaccination levels can suppress near-term outbreaks and delay the second wave. Additionally, hybrid immunity has limited impact on future COVID-19 waves with immune-escape variants.

**Conclusions:**

Enhanced understanding of the interplay between infection and vaccine exposure can aid anticipation of future epidemic activity due to current and emergent variants, including the likely impact of responsive vaccine interventions.

**Supplementary Information:**

The online version contains supplementary material available at 10.1186/s12879-024-09282-4.

## Background

The global spread of SARS-CoV-2, causing COVID-19 disease, has fundamentally changed society. Multiple epidemic waves have been experienced, beginning with the wild-type virus in early 2020, and followed by the emergence of variants such as Alpha (B.1.1.7), Delta (B.1.617.2), and more recently Omicron (B.1.1.529, subvariants BA.1, BA.2, BA.3, BA.4, BA.5 and descendent lineages) [1]. In the absence of specific preventive or disease modifying agents, the only initially effective measures to reduce the impact and burden of COVID-19 were case and contact management, restrictions to limit the number of social interactions and personal protective behaviours to reduce the per-contact likelihood of transmission. The initial hope was that vaccines might provide long lasting immunity against infection, with definitive impacts on transmission. However, while efficacy against severe disease appears to be relatively robust and long lasting, efficacy against infection acquisition and onwards transmission are lower and shorter lived—especially against the Omicron variant [2]. In response to repeated epidemic waves in highly immune populations, one or more booster doses have been recommended for sustained protection [3]. Omicron and its subvariants have caused high levels of exposure in multiple populations world-wide, even in highly vaccinated populations [4], and has led to complex “immune landscapes” across the world with varying levels of so-called “hybrid immunity” [5] in vaccinated and infected populations, consisting of immunity derived from both past infection and past vaccination.

Both infection and vaccination provide immunity boosting effects to individuals [6–13] and indirect benefits to others in the population, regardless of vaccination status, through reduction in onwards transmission [14]. This immunity directly reduces the risk of infection, and, if infection does occur, reduces disease severity and lowers onward transmission. Neutralising antibody titres, which are boosted by exposure to infection and/or vaccination, are correlated with efficacy against clinical endpoints of infection, symptoms and severe disease outcomes [15–17]. However, infection and vaccination can give different levels of protection [18], which are variable across demographics (especially with respect to age) [19]. Increased breadth and duration of antibody responses have been observed in individuals who have been both infected and vaccinated, termed “hybrid immunity” [20–25]. Crucially though, all forms of immunity *decay over time*, with additional complexity arising from observations of differential antibody waning following infection, vaccination or a combination of the two [26, 27]. Furthermore, the recent Omicron subvariants have shown immune escape in relation to immunity derived from past infections and vaccinations [2, 28–31], and even infection from earlier Omicron subvariants (BA.1, BA.2) has been shown to have reduced protection against later Omicron subvariants (BA.4, BA.5) [32].

Overall, populations have hybrid immunity, with multiple groups of individuals with different vaccination and past-infection statuses. It is a challenging process to synthesise information about individual-level immunity to population-level protection and make predictions about the severity of future COVID-19 waves and optimisation of primary and booster vaccine allocation [33–37]. Modelling has been used to support decision making around the world in regards to managing COVID-19 [38–40]. It has been used extensively to compare different vaccination strategies [41–43], but many either do not include waning of immunity [44], or do not take a hybrid-immunity approach [45]. However, the inclusion of both of these factors is key in understanding the combined population-level effect of vaccination and prior exposure upon future transmission dynamics [34, 46]. Furthermore, two populations with apparently similar levels of past-infection and vaccination coverage could still have different responses, as *epidemic history*, including the SARS-CoV-2 strain(s) and exposure sequence, also play an important role [47]. Hence, different populations and regions with unique epidemic histories require individual analysis.

In this study, we focus on dynamics of immunity in the World Health Organization (WHO) Western Pacific Region (WPR). Prior to the rollout of vaccination, the WPR region had low seroprevalence compared to other regions such as Europe [48]. As such, infection-derived immunity within this region is now largely a result of the recent Omicron wave, with relatively few infections caused by previous variant strains, unlike the populations with high prior Delta exposure pre-Omicron [34, 44]. Hence, our focus will be on analysing scenarios where a population’s past infection-derived immunity is due to Omicron.

We use an agent-based (individual-based) model of COVID-19 to consider how combinations of vaccination coverage and prior Omicron infection exposure can protect a population from future near-term Omicron waves, in the context of fixed public health measures (or lack thereof), no TTIQ (test, trace, isolate, quarantine), and with a constant vaccination capacity. We focus on the interplay between younger and older population demographics—as a proxy for two populations with different high-risk group sizes—along with a range of vaccination coverages and prior infection rates. Understanding hybrid immunity is necessary to develop efficient allocation of vaccine resources to achieve equity of outcomes across different populations with unique hybrid immunity statuses, and will also allow us to understand larger-scale future societal impacts, such as worker absenteeism and overloaded health systems. Given that past infection and vaccination provide unequal levels of immunity, we aim to identify the most advantageous strategies to protect populations from infection and severe disease outcomes in future, taking into account the current population immunity profile.

## Methods

**Fig. 1:**
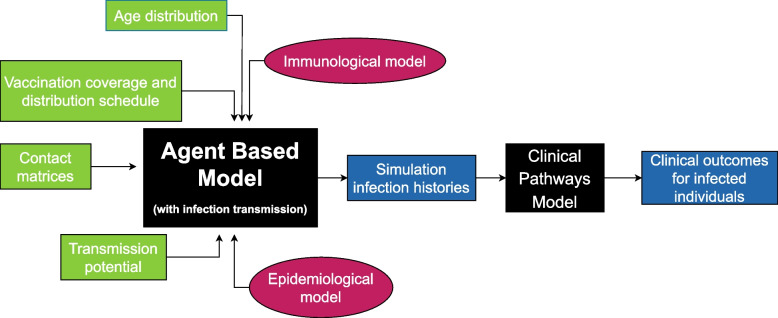
Diagram of overall simulation procedure. The core of the simulation uses an agent-based model with an underlying infection transmission model, with multiple primary inputs including immunological parameters and scenario-demography setups. The outputs are then fed into a clinical pathways model that produces clinical outcomes for infected individuals

The overall simulation procedure is comprised of an infection transmission/dynamics model that is linked to a mechanical agent-based model (cf. Refs. [37, 49] and Conway E, Lydeamore M, Walker C, et al: Optimal timing of booster doses in a highly vaccinated population with minimal natural exposure to COVID-19, in preparation). Outputs from this model feed into a clinical pathways model. The simulation process is depicted in Fig. 1, with individual model components described in more detail below.

### Agent-based transmission model

We use the agent-based COVID-19 model of Conway et al. [37, 49]. In the agent-based model, each individual has a specific age, unique neutralising antibody titre, vaccination history and own compartment label (susceptible/exposed/infected). At the beginning of each simulation, all individuals have zero neutralising antibody titre. An individual’s neutralising antibody titre can be boosted due to infection (starting from day 225) and/or vaccination (starting from the first day) and decay over time, with different levels of boosting depending on vaccine product and infection variant.

In the model, infection transmission occurs due to contact between infectious and susceptible individuals, which is dependent on input contact matrices and the individuals’ ages. Once infected, the model randomly samples to determine whether an individual is asymptomatic or symptomatic, as well as generating when they become infectious, time of symptom onset, time of isolation (if isolation or quarantine is included), and time of recovery, based on parameter values in Table 1. In each simulation, this information can be exported as a line list of infections.

**Table 1:** Model parameters. These parameters were derived by Conway et al. [37, 49] using a range of studies and the package [50], with further details available in Ref. [51]. Note that the age brackets for probabilities of symptomatic infection, relative infectiousness once infectious, and susceptibility to becoming infected upon contact with an infected individual correspond to [0, 5, 10, 15, 20, 25, 30, 35,40, 45, 50, 55, 60, 65, 70, 75, 80]

Parameter: description	Value
$\mu _{\text {AZ1}}^{0}$: $\log _{10}$ of the mean neutralising antibody titre after the first dose of AstraZeneca (no infection) [used in Eq. (1)]	-0.530
$\mu _{\text {AZ2}}^{0}$: $\log _{10}$ of the mean neutralising antibody titre after the second dose of AstraZeneca (no infection) [used in Eq. (1)]	-0.120
$\mu _{\text {PB2}}^{0}$: $\log _{10}$ of the mean neutralising antibody titre after the second dose of Pfizer (no infection); also the mean titre after one AstraZeneca dose and one infection [used in Eq. (1)]	0.154
$\mu _{\text {B}}^{0}$: $\log _{10}$ of the mean neutralising antibody titre after the first mRNA booster dose (both with and without infection); also the mean titre after two AstraZeneca doses and one infection [used in Eq. (1)]	0.323
$\mu _{U}^{0}$: $\log _{10}$ of the mean neutralising antibody titre after infection whilst unvaccinated [used in Eq. (1)]	0
$\sigma$: standard deviation of the $\log _{10}$ of neutralising antibodies across the population	0.465
$c_{h}$: midpoint of logistic function Eq. (5) of protection against hospitalisation	-1.22
$c_{d}$: midpoint of logistic function Eq. (5) of protection against death	-1.18
$c_{\xi }$: midpoint of logistic function Eq. (5) of protection against acquisition	-0.472
$c_{\tau }$: midpoint of logistic function Eq. (5) of protection against transmission	0.0295
$c_{q}$: midpoint of logistic function Eq. (5) of protection against symptomatic disease	-0.644
$\log \left( k\right)$: governs the logistic curve steepness relating antibodies to protection against disease outcome [c.f. Eq. (5)]	1.69
$k_{a}$: decay rate of neutralising antibodies [c.f. Eq. (2)]	0.00824
$\log _{10}\left( f_{\text {Omicron}}\right)$: $\log _{10}$ of the fold change in neutralising antibody titre between Delta and Omicron (BA1-like) [c.f. Eq. (4)]	-0.692
$\log _{10}\left( f_{\text {Omicron-escape}}\right)$: $\log _{10}$ of the fold change in neutralising antibody titre between Delta and the BA4/5-like immune escape variant [c.f. Eq. (4)]	-1.18
baseline age-group specific probability of symptomatic infection if infected	[0.29, 0.29, 0.21, 0.21, 0.27, 0.27, 0.33, 0.33, 0.4, 0.4, 0.49, 0.49, 0.63, 0.63, 0.69, 0.69, 0.69]
baseline age-group specific relative infectiousness once infected	[0.799, 0.688, 0.675, 0.756, 0.918, 0.965, 0.947,0.932, 0.934, 0.940, 0.954, 0.982, 1.0, 0.998, 0.990, 0.974, 0.944]
baseline age-group specific susceptibility to becoming infected upon contact with an infected individual	[0.301, 0.367, 0.433, 0.527, 0.764, 0.924, 0.983, 0.974, 0.932, 0.915, 0.929, 0.962, 1.0, 0.972, 0.882, 0.824, 0.802]
$R_{0}$ ratio between the original Omicron variant (BA1/2-like) and the BA4/5-like immune escape variant (the added transmissibility of the immune escape variant)	1.3

### Clinical pathways model

We use the stochastic COVID-19 clinical pathways model by Conway et al. [37, 49]. The clinical pathways model relates demographic information and neutralising antibody titre to clinical outcomes. For each symptomatic infection occurrence, the clinical pathways model takes in age and neutralising antibody titre at time of infection of the infected person and generates a clinical trajectory for each symptomatic infection, including whether the individual will require hospital admission, or will die.

Note that the clinical pathways model is independent of the main agent-based model described above, which allows for greater flexibility in the overall simulation process and also allows the clinical pathways model to be used for multiple epidemic models. However, due to this independence, it is possible that patients who die, according to the clinical pathways model, remain in the agent-based simulation. Due to the low number of deaths, relative to infections, this should not have major effect on the two models’ outcomes [37, 49].

### Immunological model

Neutralising antibodies are one of the many biomarkers associated with COVID-19 immunity [18, 52, 53], so we use them to regulate each agent/individual’s interaction with the SARS-CoV-2 virus, including each individual’s level of protection against infection and severe clinical outcomes [15, 16, 50]. In particular, we use the model from [15, 16] to use neutralising antibodies to determine each individual’s level of protection against infection, symptomatic disease, onward transmission given breakthrough infection, hospitalisation and ICU admission, and death. The antibodies follow exponential decay over time, with our model assuming that the decay rate is the same across all forms of initial antibody boosting (whether vaccination or prior infection).

When an antibody boosting event occurs, the individual gets a new antibody titre, $a^0$, that is sampled from:

1 $$\begin{aligned} \log _{10}(a^0) \sim \mathcal {N}(\mu _j^x,\sigma ^2), \end{aligned}$$

where $\mu _j^x$ is the mean neutralising antibody titre against strain *x* (Delta or Omicron) after boosting process *j* (infection or vaccination), and $\sigma ^2$ is the variance of antibodies across the population (Table  1). Note that infection prior or post vaccination results in the same titre. The highest neutralising antibody titre boosting occurs with the booster dose, and does not rise with extra infections. There is an upper limit on titre, which is equivalent to either two vaccinations and an infection, or three vaccinations. (For further details on how the titres for determined for combinations of infection and vaccination, see Table 1 of [49].) We also assume that the neutralising antibodies decay exponentially over time *t* after an exposure event:

2 $$\begin{aligned} \log _{10}a_i(t) = \log _{10} (a_i^0) - \frac{k_a}{\log (10)}t. \end{aligned}$$

Note that there is an additional scaling as many of the parameters in Table 1 are for strain 0, i.e. the Delta strain. To convert to neutralising antibodies against the Omicron strain, the fold change $f_\text {Omicron}$ is used:

3 $$\begin{aligned} \mu _j^\text {Omicron} = \mu _j^0 + \log _{10}(f_\text {Omicron}). \end{aligned}$$

To convert an individuals’ Omicron BA.1 titres, $a^{Omicron}$, to titres against the BA.4/5-like variant:

4 $$\begin{aligned} \log _{10}(a^{\text {Omicron-escape}}) = \log _{10}(a^{\text {Omicron}}) - \log _{10}(f_\text {Omicron}) + \log _{10}(f_\text {Omicron-escape}). \end{aligned}$$

The immunological model parameters are drawn from Refs. [37, 49, 50] (Table 1). The model parameters broadly follow the characteristics for the Omicron BA.1 subtype. We include two additional parameters in the case of an Omicron BA.4/BA.5-like immune escape variant: 1) $R_{0}$ ratio between the original Omicron variant (BA1/2-like) and the BA4/BA5-like immune escape variant to account for an inherent increase in transmissibility; and 2) $\log _{10}\left( f_{\text {Omicron-escape}}\right)$ to account for an approximately three-fold reduction in neutralising antibody protection derived from preceding immunising exposures [50, 54].

The relationship between neutralising antibody titre $a_{i}$ of an individual to protection against some disease outcome $\rho _{\alpha }$ (hospitalisation, death, acquisition etc.) is [37, 49]:

5 $$\begin{aligned} \rho _{\alpha }=\frac{1}{1+\exp \left[ -k\left( \log _{10}\left( a_{i}\right) -c_{\alpha }\right) \right] }, \end{aligned}$$

where *k* determines the steepness and $c_{\alpha }$ corresponds to the midpoint of the logistic function for a particular disease outcome ($\alpha$ = hospitalisation, death etc...) (see Table 1). The protection against disease outcomes is then used to calculate the probabilities of obtaining a disease outcome, i.e., the probability of being infected, of being hospitalised, and so forth, with the baseline probabilities given in Table 1. See Conway et al. [37, 49] and the supplementary code base for more details.

### Fixed input parameters

In the WPR scenarios we consider, the following components are fixed:

**Vaccine type**: We assume that all primary doses are ChAdOx1 nCoV-19 (AstraZeneca) and booster doses are BNT162b2 (Pfizer/BioNTech).

No **TTIQ **(Test, Trace, Isolate and Quarantine). This means there are no public health measures in these scenarios.

**Fig. 2:**
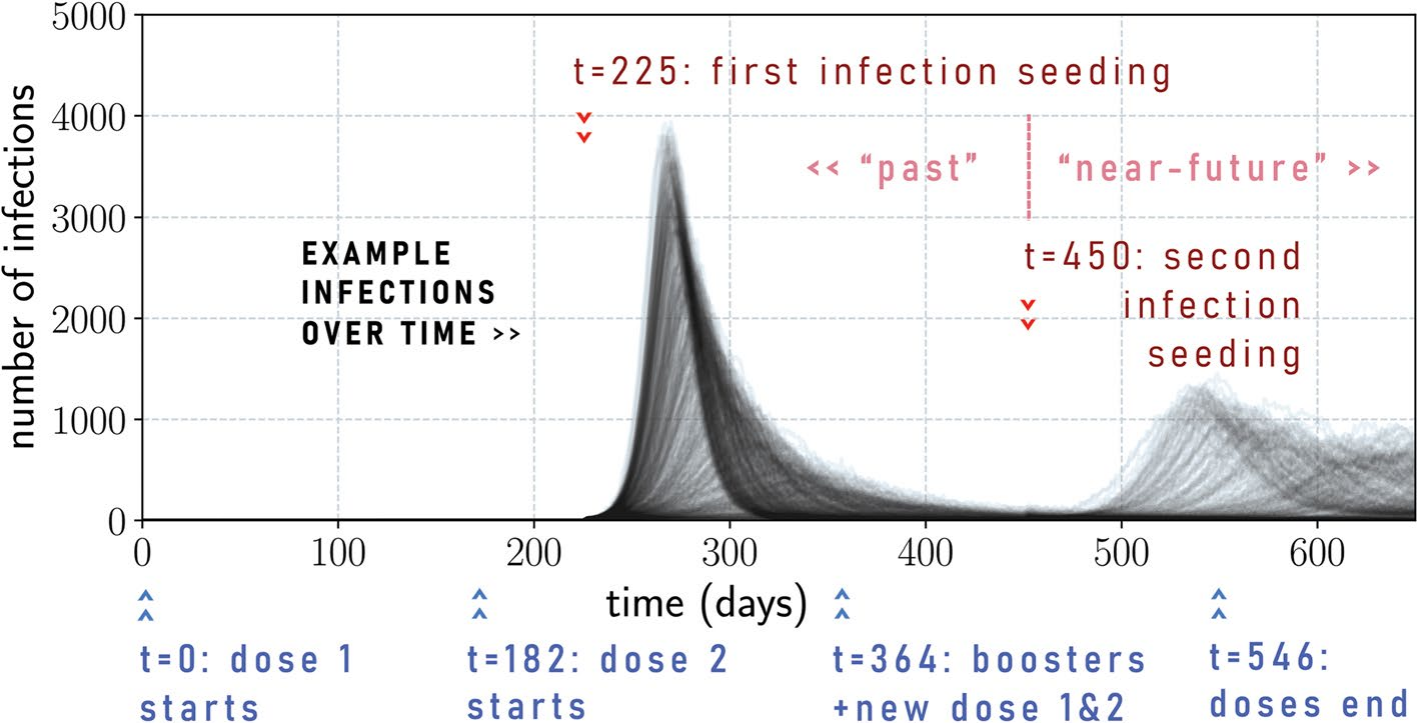
Timeline of vaccination schedule and infection seedings with examples of infection time series. There is a vaccination rollout that occurs in three consecutive stages, starting at $t=0$, $t=182$, and $t=364$ and ending at $t=546$. The first wave and the second wave are generated by randomly seeding 100 infections in the population (which could occur due to a super-spreader event, for example) at times $t=225$ and $t=450$. The example time series are for a second wave due to the same variant as the first wave

**Timeline**: We assume a fixed schedule for vaccination and the seeding of infection (depicted in Fig. 2).

There are three vaccination stages that take 26 weeks each, i.e., 6 months each: the first dose stage, the second dose stage, and the mixed booster and new primary course stage. In the first stage, first doses are given to the allocated population, and in the second stage, the second dose is given to the same groups. Within the first and second stages, the $65+$ age group always gets their first dose or second dose first, followed by randomly assigned vaccinations among the $5-64$ age group. At the end of the second stage, $V\%$ of the population have received the primary course, where $V=20,50,80$. In the third stage, $80\%$ of the fully vaccinated population receive a booster dose, while remaining available doses are given out as new primary doses. An example of this is given in Fig. A4 in the Supplementary Material 1. The duration of 6 months is based on the general principle of having 4–6 months between primary doses and booster doses [55].

We seed two infection waves with 100 infections each: one at time $t=225$ (approximately the 32nd week, or over seven months), such that the first wave is largely over by the end of the second vaccination stage, and one at time $t=450$, where protection from the first wave has waned enough such that a second wave could possibly occur. Any infection that occurs before $t=450$ is marked as a “past” infection, while anything after $t=450$ (and before $t=650$) is marked as a “near-future” infection.

The first infection wave timing is based on what happened in the Western Pacific Region: COVID-19 vaccines were available in early 2021 (for example, February 2021 in Australia and Japan [56, 57]; and April 2021 in Samoa and Papua New Guinea (to members of the public) [58, 59]); meanwhile, the Omicron variant emerged in November 2021 [60]. That is, there was at least seven months between the start of vaccination before the Omicron variant arrived. This first wave ensures that our simulated populations have “past” hybrid immunity by $t=450$.

Note that we end our analysis at time $t=650$, which is approximately three months after the final vaccination stage finishes. This allows time for the earlier vaccination to take some effect. We do not run simulations for any extra time until all second waves are finished in situations where the second seeding does not spark a second wave immediately. This is because if we do continue to run the simulation, immunity will continue to wane, and in some situations, we would see a third wave. Furthermore, we would expect that some populations would continue a fourth vaccination stage and so forth. In the Supplementary Material Fig. A9, we explore ending the analysis earlier versus later. We find that if we end the analysis too early, there is little differentiation between different vaccination coverages, while if we end the analysis later, our conclusions do not change.

### Scenarios

The scenarios and demographics that we change across different simulations are as follows.

**Fig. 3:**
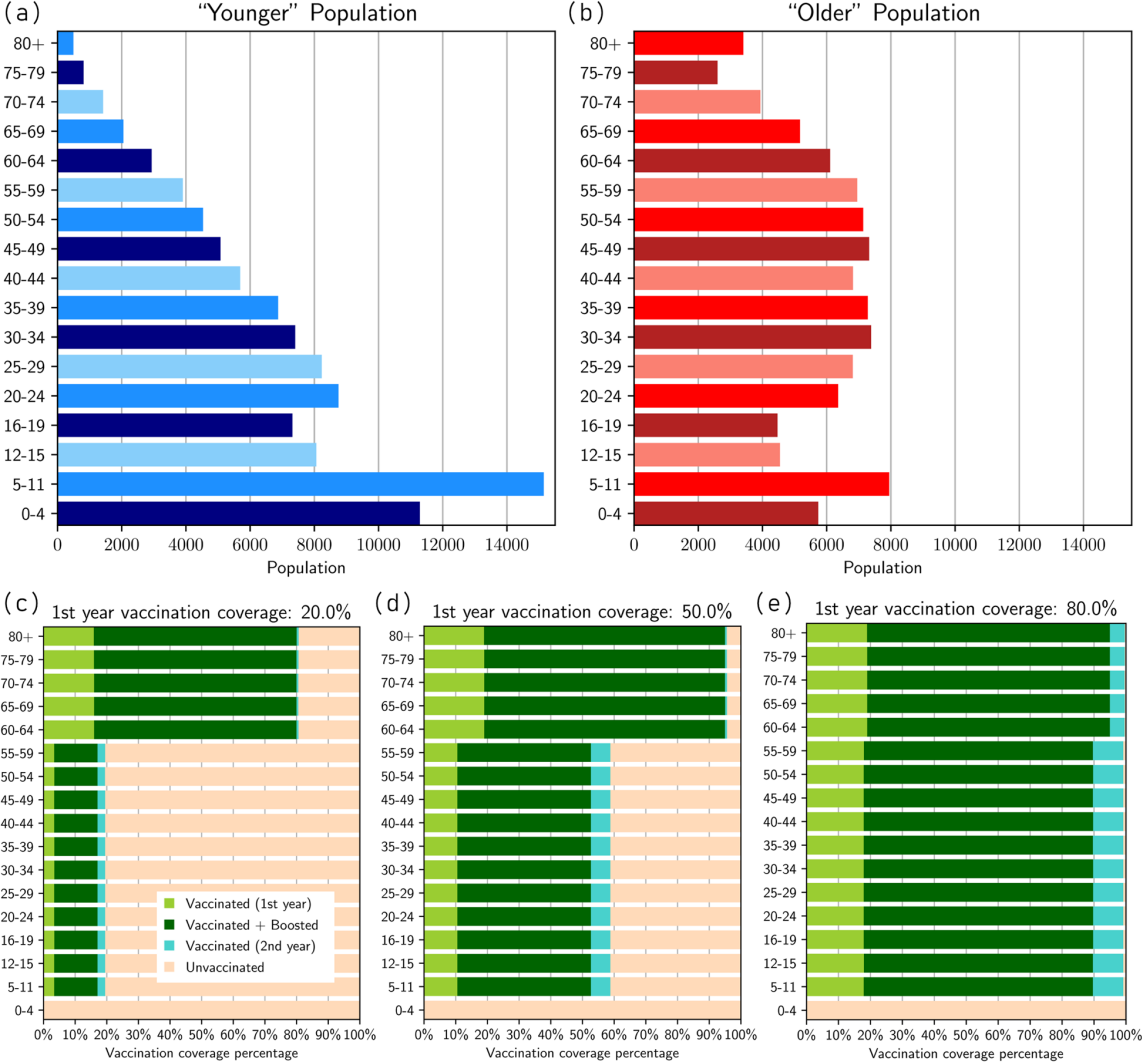
Scenarios considered: **a** exemplar“younger” population demographics, **b** “older” population demographics (see Appendix A of the Supplementary Material 1 for the construction of these exemplar populations), **c** 20% vaccination coverage, **d** 50% vaccination coverage, **e** 80% vaccination coverage, where the coverage value corresponds to primary vaccination coverage by time $t=364$. Note that **c**, **d**, **e** detail the proportions in the younger population; the proportions in the older population are similar

**Population age demographics**: see Fig. 3a and b. We generate populations with 100,000 people. We consider “older” and “younger” populations, which are derived from averaged age-proportions across the majority of countries in the Western Pacific regions, using the population data acquired through  UN World Population Prospects [61] for the year 2021. We defined “older” countries as those with an $OADR\ge 15$ and “younger” countries as having an $OADR\le 12$, where OADR is the *old-age to working-age demographic ratio*, which we calculate as a ratio between the +65 year old population and the 20-64 year old population. The individual country-level data used can be seen in Figs. A1 and A2 of the Supplementary Material 1.

Additionally, each older/younger demographic has a different **contact matrix**, which governs the social mixing (and thus infection spread) in the model. To derive some exemplar contact matrices, we used aggregated contact matrices from “older” ($OADR\ge 15$) and “younger” ($OADR\le 12$) countries that could be found on socialcontactdata.org [62–72] (where for “older” countries we included Belgium, Finland, France, Germany, Hong Kong, Italy, Luxembourg, Poland; and “younger” included Viet Nam and Zimbabwe. These countries fell into the appropriate OADR values and had contact matrix values for all the age-groups we required.)

**Vaccination coverage**: see Fig. 3c, d, e, and f. The vaccine allocation broadly follows WHO guidelines [3], which recommends prioritising the vaccination of older and higher-risk groups. Aside from the zero-vaccination scenario, either $20\%$, $50\%$, or $80\%$ total vaccination coverage is achieved at the end of the second stage, i.e. $t=364$, reflecting differing levels of vaccine access/health sector capacity. At the lower vaccination rate of $20\%$, we first allocate doses such that $80\%$ of the $60+$ age group are fully vaccinated by the end of the first two stages. At the higher vaccination rates of $50\%$ and $80\%$, we allocate initial doses such that $95\%$ of the $60+$ age group are fully vaccinated by the end of the first two stages—a fully vaccinated cohort is unrealistic. The remaining available doses in the first two stages are then equally (proportionately) allocated to the 5–59 age groups. In the third stage, $80\%$ of all vaccinated individuals are allocated booster doses (no prioritisation of any age groups), with the remaining doses given equally across the age-groups to unvaccinated individuals. An example of this is given in Fig. A5 of the Supplementary Material 1.

**Second wave variant type**: We considered scenarios with either a second wave caused by the same Omicron variant as the in the first wave (default scenario), or with a *new* immune escape variant. The variant in the first wave is broadly referable to the characteristics of the Omicron BA.1 variant. The immune escape variant is Omicron “BA.4/5-like”, as it is currently one of the variants of most global concern. The immunological parameters were determined using the methods described in  [50] (also see the methods described in [37, 49]).

**Inherent transmissibility** of the variant in that particular population: Different countries do not have the same $R_{eff}$ or $R_{0}$ for the same virus. Furthermore, changes in social mixing patterns, due to social distancing measures for example, will change the population level of transmission. Therefore, rather than fixing the transmissibility across all simulations, we *vary* it ($R_{0}\in$[0.85, 0.9, 0.95, 1., 1.05, 1.1, 1.15, 1.2, 1.25, 1.3, 1.35, 1.4, 1.45, 1.5, 1.55, 1.6, 1.65, 1.7, 1.75, 1.8, 1.85, 1.9, 1.95, 2.0, 2.05]), which gives rise to different attack rates in the population. We assume that the populations act the same during both waves (at whatever level of restrictions they may have). If the second wave is due to the same COVID-19 variant, then the transmissibility remains the same, i.e., within any individual simulation, the level of transmission is constant over time. If the second wave is due to an immune-escape variant with increased transmissibility, then the original transmissibility is multiplied up by the value given in Table 1.

For each scenario and set of input parameters, we ran 10 simulations, each producing a single infection history with information about the number and times of infection and vaccinations for each individual in the population. From each individual simulation, our stochastic clinical model produces five alternative clinical pathway histories, with information about number of infections, severe cases, hospital admissions, ICU admissions, and deaths per day. We aggregated the total cases, ICU admissions, and deaths per day for each simulation. We calculated attack rates as the number of infections divided by the population size. The “past attack rate” includes infections between day 0 and day 449 inclusive, while the “future attack rate” includes infections on days 450 to 649 inclusive.

## Results

**Fig. 4:**
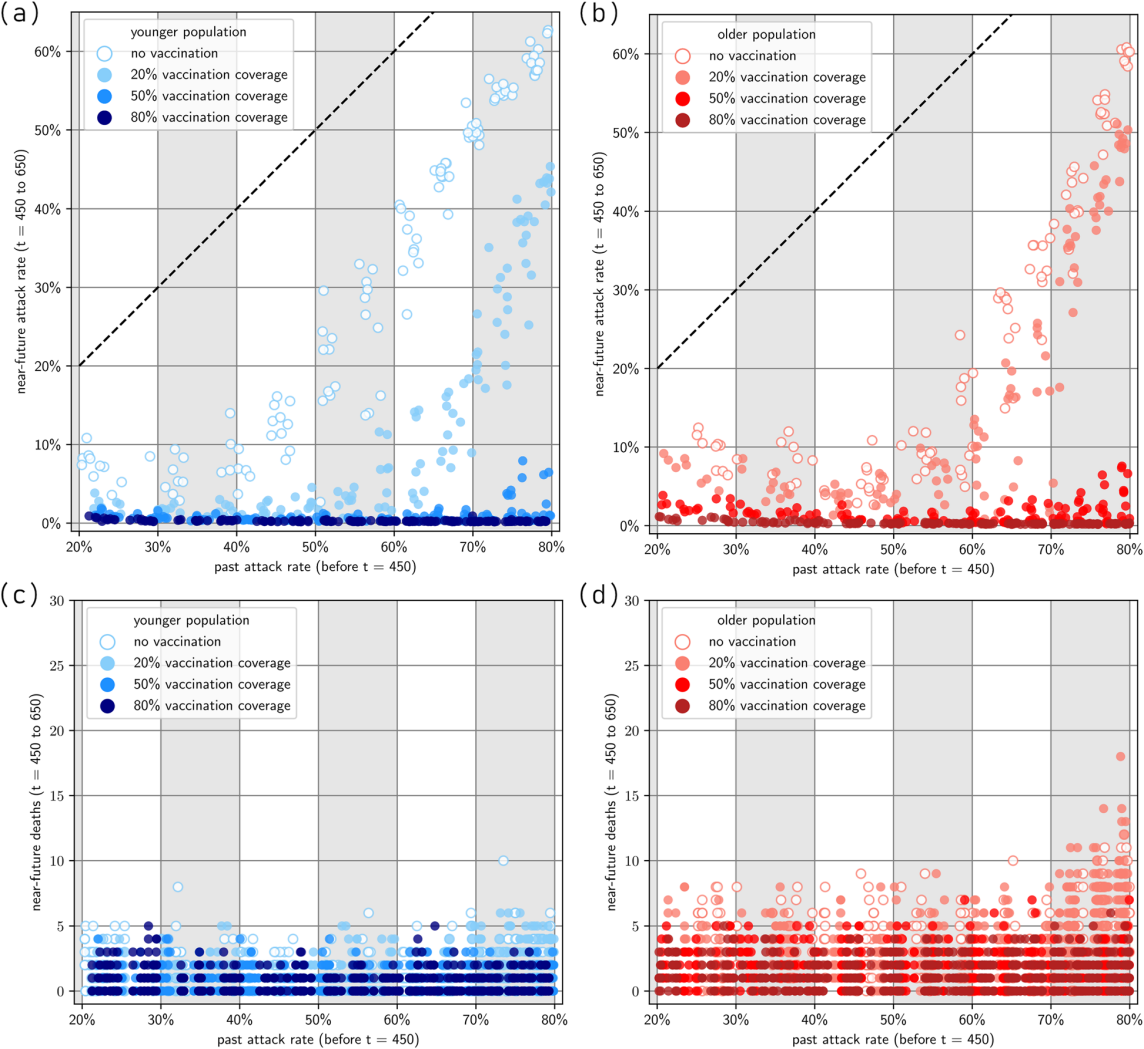
Near-future outcomes given past immunity. **a** Younger population, near-future attack rate; **b** Older population, near-future attack-rate (the diagonal line represents where past and near-future attack rates are equal); **c** Younger population, near-future deaths; **d** Older population, near-future deaths. Note that past attack rate is calculated between $t\in \left( 0,450\right)$. Past attack rate is dependent on transmission potential, which is different for various simulations, reflecting different populations’ intrinsic transmission. Near-future attack rate and near-future deaths are calculated between $t\in \left[ 450,650\right)$. Note that we have only included simulation results in which the past attack rate is between 20% and 80%

**Fig. 5:**
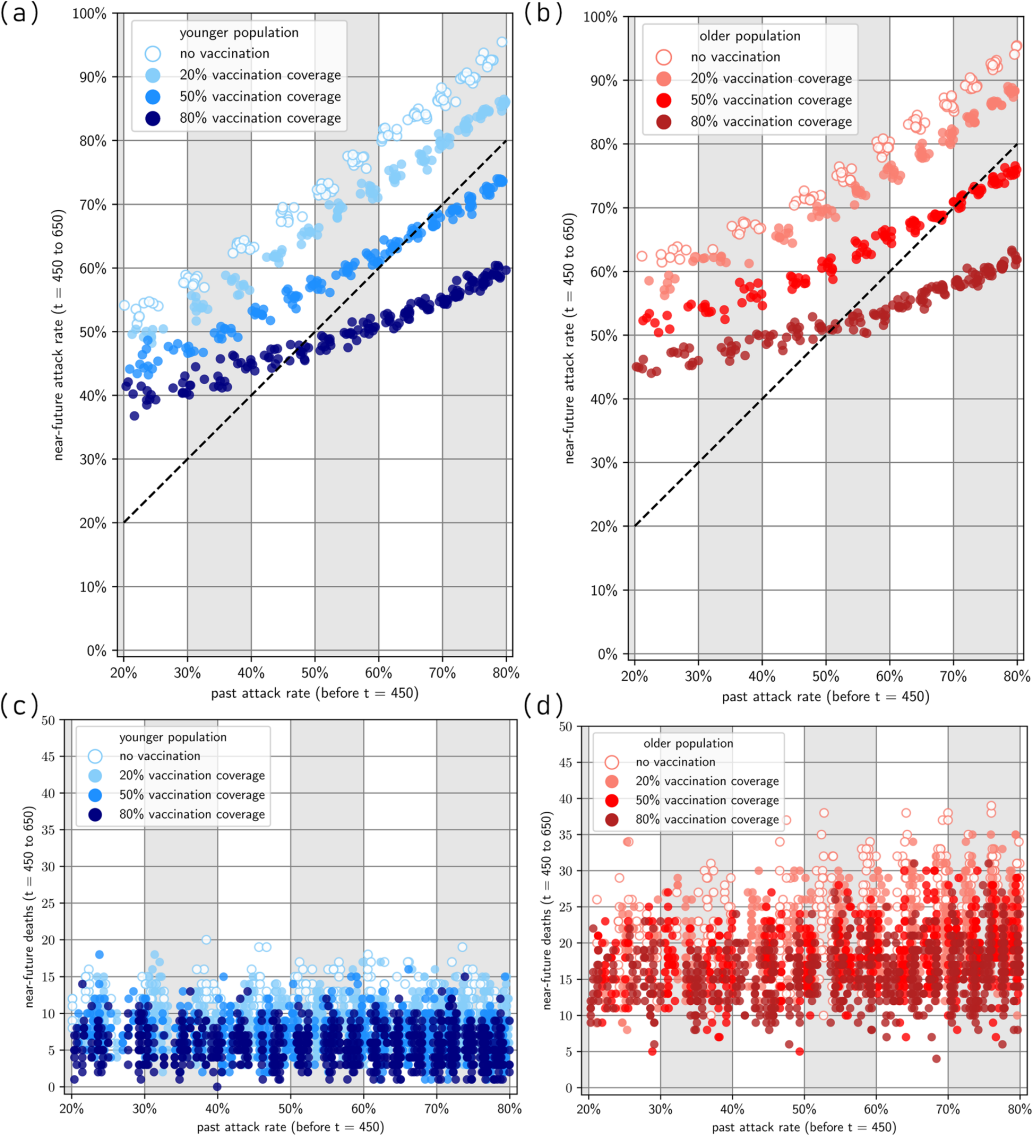
Near-future attack rate and deaths given past immunity, for a second wave due to a BA.4/BA.5-like immune escape variant. The diagonal line represents where past and near-future attack rates are equal. Note that we have only included simulation results in which the past attack rate is between 20% and 80%

Figure 4 presents results for the scenarios where both waves are due to the same Omicron BA.1-like variant. Figure 5 presents results for the case with the immune-escape variant during the second wave. Extended figures are given in Appendix A.3 of the Supplementary Material 1.

### Reinfection with the same Omicron BA.1-like variant

In the absence of vaccination, immunity from infection alone wanes rapidly enabling a second wave. Assuming that the variant characteristics are unchanged, we observe a consistent linear relationship between the size of the first and second wave, especially at transmissibility values that lead to a first-wave attack rate of 50% or above. At high attack rates, the immunity derived from the first wave only gives a modest constraint on the size of the second wave (for example, a past attack rate of 80% during the first wave leads to an attack rate of 60% in the second wave) (Fig. 4 a, b).

There is little difference in the protection provided by different vaccination coverage levels when the past attack rate is $50\%$ or lower. The past attack rate size is largely determined by the inherent transmissibility of the Omicron variant in the population. This means that for a lower past attack rate, the virus is less transmissible in that population, due to some inherent properties of the population. In countries with lower transmission, high vaccination is not as important provided that only the same variant is in circulation (Fig. 4).

For scenarios where the past attack rate is higher than $50\%$, we observed that different vaccination coverage levels have noticeable effects. Increasing vaccination coverage from 20% to 50% can delay the next wave in the immediate future. If vaccination coverage is only 20% then a high past attack rate is insufficient to ensure a low future resurgence of infections with the same variant. This is most likely due to the high intrinsic disease spread which caused the high past attack rate and similarly caused the high near-future attack rate, as well as the short-lived nature of immunity derived only from natural infection.

We observed little difference in total infections at vaccine coverage levels between $50\%$ and 80%. This could be due to the fixed assumption that for each of these coverage scenarios, takeup within the 65+ age group is the same (at 95% in-group coverage). In terms of more severe clinical outcomes such as death, older populations have consistently worse clinical outcomes than the example younger population. Furthermore, a very high vaccination rate of 80% does have noticeably better clinical outcomes than 50%, especially in older populations. Since the high-risk coverage is the same in both scenarios, this indicated that there is benefit to vaccinating low-risk groups (Fig. 4 c, d).

### Reinfection with an Omicron BA4/BA5-like immune escape variant

In general, we found that for scenarios where the second wave is dominated by the Omicron BA.4/BA.5-like immune escape variant, the previously observed protective effects of vaccination against the second wave were substantially reduced, as the efficacy against transmission and severe disease is impacted by the immune escape. For these immune escape scenarios, we observed a linear relationship between past attack rate and future attack rate. Furthermore, future attack rates are often *higher* than past attack rates (Fig. 5 a, b).

We found that older populations have slightly greater near-future attack rates given the same past hybrid immunity as younger populations, but overall we did not observe a substantial difference between peak sizes, similar to the previous scenarios where transmission is dominated by a single viral strain. For severe outcomes such as death in the second wave, we found that the protective effect of vaccination was substantially reduced, with much smaller differences in clinical outcomes between different vaccination coverages. Once again, older populations (i.e. populations with larger high-risk groups) experienced consistently worse clinical outcomes than the younger populations (Fig. 5 c, d).

## Discussion

The Omicron variant of COVID-19 has spread across the world. Unlike previous variants, Omicron has increased transmissibility and immune escape [2, 28–31], meaning that both vaccination and past infection confer a lower level of protection against Omicron (re)infection. We considered different populations with either ‘older’ or ‘younger’ demographics and with varying existing hybrid immunity. These populations were then subjected to a second wave of either the same prior variant (BA.1 like) or a different immune escape variant (BA.4/BA.5 like). We find that high vaccination coverage makes a noticeable difference in reducing the number of infections and severe outcomes in populations. Additionally, a population with a high past attack rate—reflecting a society with certain mixing and environmental factors that increase disease spread—is likely to have a high future attack rate and will benefit from higher vaccination coverage. In contrast, if populations with a low past attack rate—reflecting different intrinsic society mixing and limited disease spread, including potential public health measures—continue to behave the same going forward, then high vaccination does not have high impact against a second wave.

We find that higher vaccination coverage is more important for older populations, that is, populations with larger high-risk groups. Even if older and younger populations have the same future wave size—i.e. in terms of infection numbers—the older populations consistently have worse clinical outcomes. Vaccination is important in reducing severe outcomes, and thus is important in populations at higher risk of severe outcomes. This work highlights that populations with large high-risk groups must do more to protect themselves in order to achieve the same outcomes compared to those populations with smaller high-risk groups.

We also found that high vaccination coverage can *delay* the emergence of a second wave. This delay is important because a longer time between waves allows more time to vaccinate populations, fewer infections and deaths across the same time frame, less economic disruption, and so forth. If there is a sufficient interval between campaigns, a fourth booster dose may be able to mitigate against some of these outcomes in the older population by “resetting” short term protection to a higher level, but that has not been simulated here. For example, Hogan et al. [34] created a model to predict how protection from past infection, vaccination and boosting declines over time. They found that in partially vaccinated populations, (first) boosters should be preferentially given to high-risk/older groups instead of giving new primary doses to low-risk/younger age groups; considering waning immunity, we can expect this reasoning to apply for second and subsequent boosters.

A strength of our study is that using our agent-based transmission model, we have been able to systematically model complex exposure histories for various populations with differing overall levels of waning hybrid immunity and compare how different populations fare under various scenarios. As vaccinations and exposures increase over time, the hybrid immunity landscape of populations will become more complex, further increasing the utility of models that have the ability to capture diverse exposure and vaccination histories. Many of the earlier studies did not include flexible hybrid immunity arrangements and/or did not include waning immunity or multiple reinfections (e.g. [43–45]), which here we show is key to the occurrence, size and timing of the second wave. While we did not include heterologous vaccination schedules, such as was modelled in  [73], our modelling framework has the ability to support these scenarios.

There are several limitations to our study. First, our model did not have two variants circulating at the same time, instead using a sharp change point where all new infections following a certain time were with the immune escape strain. However, given that new variants have historically replaced currently circulating variants over relatively short time periods [74, 75], we do not envisage this would greatly change our results. Second, we did not consider the breadth of antibody response; we assume that while natural infection and vaccination boost antibodies by different amounts, the model then wanes both at the same rate instead of different rates. The next generation of vaccines will very likely be bivalent, as they are more broadly cross reactive and provide more durable protection. With all the increasing *breadth* of protection becoming important, a single antibody value may no longer be sufficient, instead requiring a multidimensional approach. Thirdly, in the absence of robust population level evidence to the contrary, our model assumes that neutralising antibody titres are the only proxy correlate of protection against both infection and clinical outcomes. However, we expect there exist other correlates of protection [76, 77], and as evidence regarding these mechanisms accrues, we will incorporate them into future model development. Additionally, we could improve the cohesion between the agent-based transmission model and the clinical pathways model, for example by embedding the clinical model within the transmission simulation, to avoid the situation where dead agents continue to become infected and transmit disease.

## Conclusions

Our study clearly shows how waning immunity and emergence of vaccine escape variants limits the impact of COVID-19 vaccines against transmission. The benefits of vaccination and past infection derived protection against COVID-19 disease depend critically on many factors, including demography, health systems capacity, vaccine efficacy, and breadth of protection, especially against new variants. Protection against severe disease is more robust, with achievable gains depend on underlying population demographics and risk. Ideally, vaccines would be allocated after assessing hybrid immunity at both the individual and population level in order to maximise the protection against future waves especially if vaccine stocks are limited. Our results suggest that populations with low vaccination coverage and high past infection rate should still consider vaccination if public health measures are not enforced or social mixing is not reduced, with particular emphasis on protecting those at higher risk, such as older age groups. In addition, our work suggests that populations with past high infection rates will likely continue to have high future infection rates, if nothing were to change. In general, given an approximate age structure, past attack rate and past vaccination coverage, our work can estimate the real world effects of the next epidemic wave, which can then be used for future planning.

The hybrid immunity landscape will only become increasingly complex as time goes on: with more circulating variants and with more vaccinations and more unique and individual exposure histories. How this can be robustly incorporated into mathematical models—especially in cases of limited available data—will be an ongoing challenge. As we move from the initial pandemic stage of COVID-19 to ongoing endemic transmission, we need tailored responses, sustainable long term protection and vaccination schedules to protect those at risk of severe disease and have equity of outcomes going forward. The role of future vaccines for resilience to ‘endemic’ disease and response to variants of concern remains to be determined for each individual country context. Our flexible framework is capable of considering different country contexts by inputting different age distributions, vaccination schedules, contact matrices and other key parameters and thus can assist in determining the relative benefits of vaccines in different populations going forward.

### Supplementary Information

**Additional file 1.** *Modelling the impact of hybrid immunity on future COVID-19 epidemic waves: Supplementary Material* (Supplementary Material.pdf) which contains extended modelling details and additional results. *Modelling the impact of hybrid immunity on future COVID-19 epidemic waves: Code* (hybrid_immunity_code.zip) which contains the source code for the agent-based transmission model and clinical pathways model.

## Data Availability

The datasets and modelling and analysis code used during this study are included in this published article as part of its supplementary information files. The data generated from the modelling code analysed during the current study are available from the corresponding author on reasonable request.

## Abbreviations

OADR: Old-age to working-age demographic ratio
TTIQ: Test, trace, isolate, quarantine
WHO: World Health Organization WHO
WPR: Western Pacific Region
