# Supplementary material for: Modelling the impact of hybrid immunity on future COVID-19 epidemic waves
Source: BMC Infect Dis. 2024 Apr 16;24:407. doi: 10.1186/s12879-024-09282-4 (PMC11020923; doi:10.1186/s12879-024-09282-4)
Supplement: Supplementary file 1 — Additional file 1. Modelling the impact of hybrid immunity on future COVID-19 epidemic waves: Supplementary Material (Supplementary Material.pdf) which contains extended modelling details and additional results. Modelling the impact of hybrid immunity on future COVID-19 epidemic waves: Code (hybrid_immunity_code.zip) which contains the source code for the agent-based transmission model and clinical pathways model. [file 12879_2024_9282_MOESM1_ESM.zip › Supplementary Material_ESM.pdf]

Modelling the impact of hybrid immunity on future COVID-19 epidemic waves: Supplementary Material

Thao P. Le[1,2,3], Isobel Abell[1,2], Eamon Conway[4], Patricia T. Campbell[5,6], Alexandra B. Hogan[7,8], Michael J. Lydeamore[9], Jodie McVernon[5,10], Ivo Mueller[4,11], Camelia R. Walker[1], Christopher M. Baker[1,2,3]

1 School of Mathematics and Statistics, The University of Melbourne, Grattan Street, Melbourne 3010, Victoria, Australia

2 Melbourne Centre for Data Science, The University of Melbourne, Grattan Street, Melbourne 3010, Victoria, Australia

3 Centre of Excellence for Biosecurity Risk Analysis, The University of Melbourne, Grattan Street, Melbourne 3010, Victoria, Australia

4 Population Health & Immunity Division, Walter and Eliza Hall Institute of Medical Research, 1G Royal Parade, Melbourne 3052, Victoria, Australia

5 Department of Infectious Diseases at the Peter Doherty Institute for Infection and Immunity, The University of Melbourne, 792 Elizabeth St, Melbourne 3000, Victoria, Australia

6 Melbourne School of Population and Global Health, The University of Melbourne, Bouverie St, Carlton 3053, Victoria, Australia

7 School of Population Health, University of New South Wales, Sydney 2033, New South Wales, Australia

8 MRC Centre for Global Infectious Disease Analysis, Jameel Institute, School of Public Health, Imperial College London, Exhibition Road, London SW7 2AZ, United Kingdom

9 Department of Econometrics and Business Statistics, Monash University, Wellington Road, Melbourne 3800, Victoria, Australia

10 Victorian Infectious Diseases Reference Laboratory Epidemiology Unit, The Royal Melbourne Hospital at the Peter Doherty Institute for Infection and Immunity, 792 Elizabeth St, Melbourne 3000, Victoria, Australia

11 Department of Medical Biology, The University of Melbourne, Grattan Street, Melbourne 3010, Victoria, Australia

Contents

A Simulation details and results **2**

A.1 Constructing the exemplar “younger” and “older” populations 2

A.2 Detailed vaccination allocation and schedule 3

A.3 Extended results 5

A.4 50% vs 80% booster coverage 7

B If vaccination efficacy was worse **7**

Bibliography **15**

A Simulation details and results

A.1 Constructing the exemplar “younger” and “older” populations

There are multiple methods to measure population aging, especially in light of increasing life expectancies and extended working age across the world [1, 2]. For our populations, we use the old-age to working-age demographic ratio, OADR, which is calculated as:

$$\text{OADR} = \frac{\text{population aged 65 and over}}{\text{population aged } 20-64} \times 100. \tag{1}$$

As mentioned in the main text, our exemplar “younger” and “older” populations are constructed from the averaged age distribution across multiple countries in the WHO-defined Western Pacific Regions. To obtain two different age distributions—which is also a proxy for size of high-risk group—we defined “younger” countries as having an $OADR \leq 12$ and “older” countries as those with an $OADR \geq 15$ (we do not include countries whose OADR lay between 12 and 15). Coupled with the international population data available at [3] for the year 2021, the countries we included in the “younger” and “older” groups were:

1. “Younger”: Mongolia, Brunei Darussalam, Cambodia, Lao People’s Democratic Republic, Philippines, Fiji, Papua New Guinea, Soloman Islands, Vanuatu, Kiribati, Micronesia (Fed. States of), Samoa, and Tonga (see Fig. A1).

2. “Older”: China, Hong Kong SAR, Macao SAR, Japan, Republic of Korea, Singapore, Australia, New Zealand, New Caledonia, Guam, and French Polynesia (see Fig. A2).

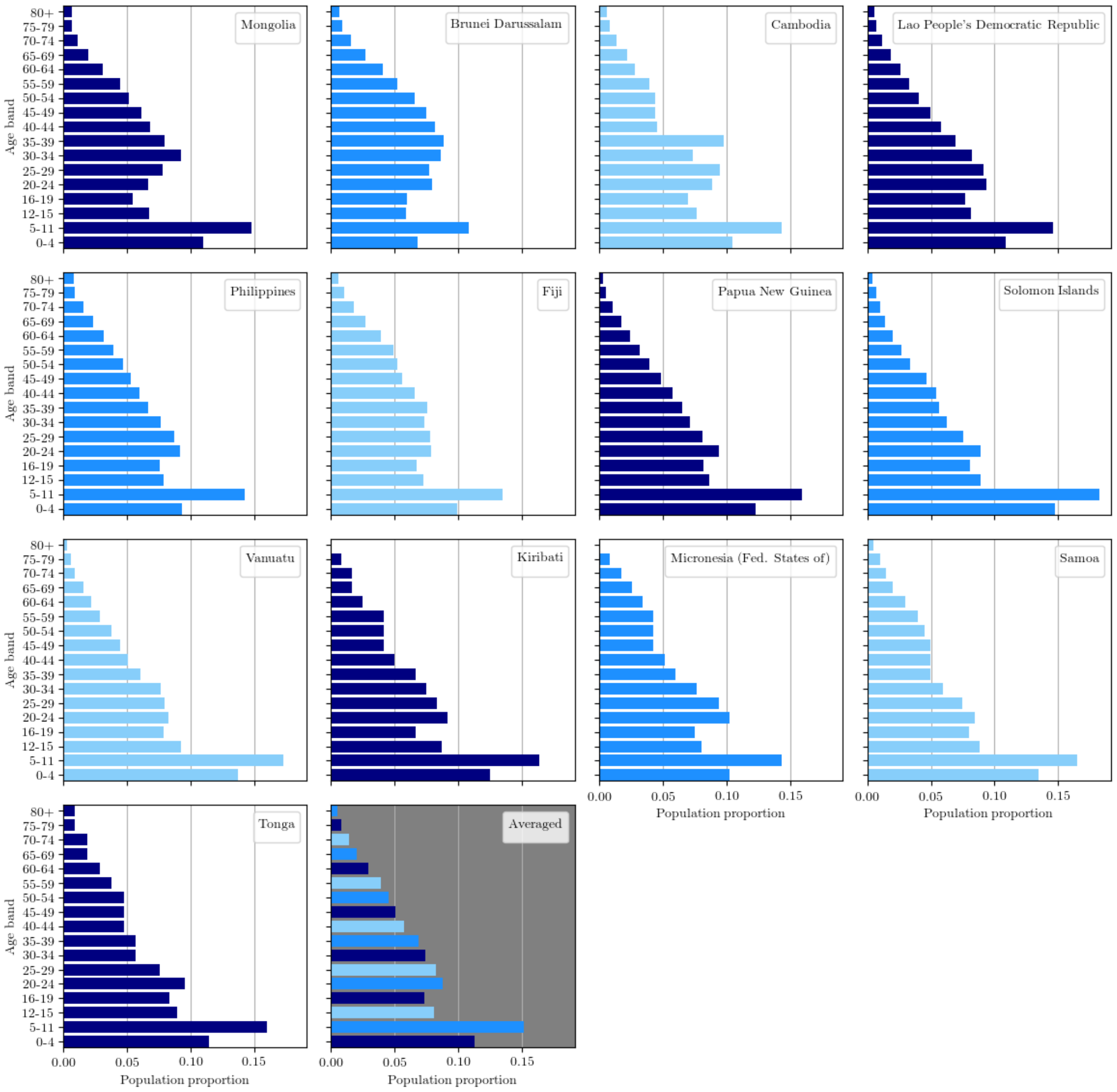

Figure A1: **Countries used to create the exemplar "younger" population distribution**: relative proportions across age groups were averaged.

A.2 Detailed vaccination allocation and schedule

There are two components to the vaccination program in our simulations: (1) the vaccine schedule, and (2) the vaccine allocation (which also defines the vaccine capacity).

The vaccine schedule has three different stages, each 26 weeks each—roughly half a year. An example of the schedule is given in Fig. A4.

1. In the first stage, first doses are given to the allocated groups that will be vaccinated during the first year. The 65+ individuals are vaccinated first, followed by randomly assigned vaccinations

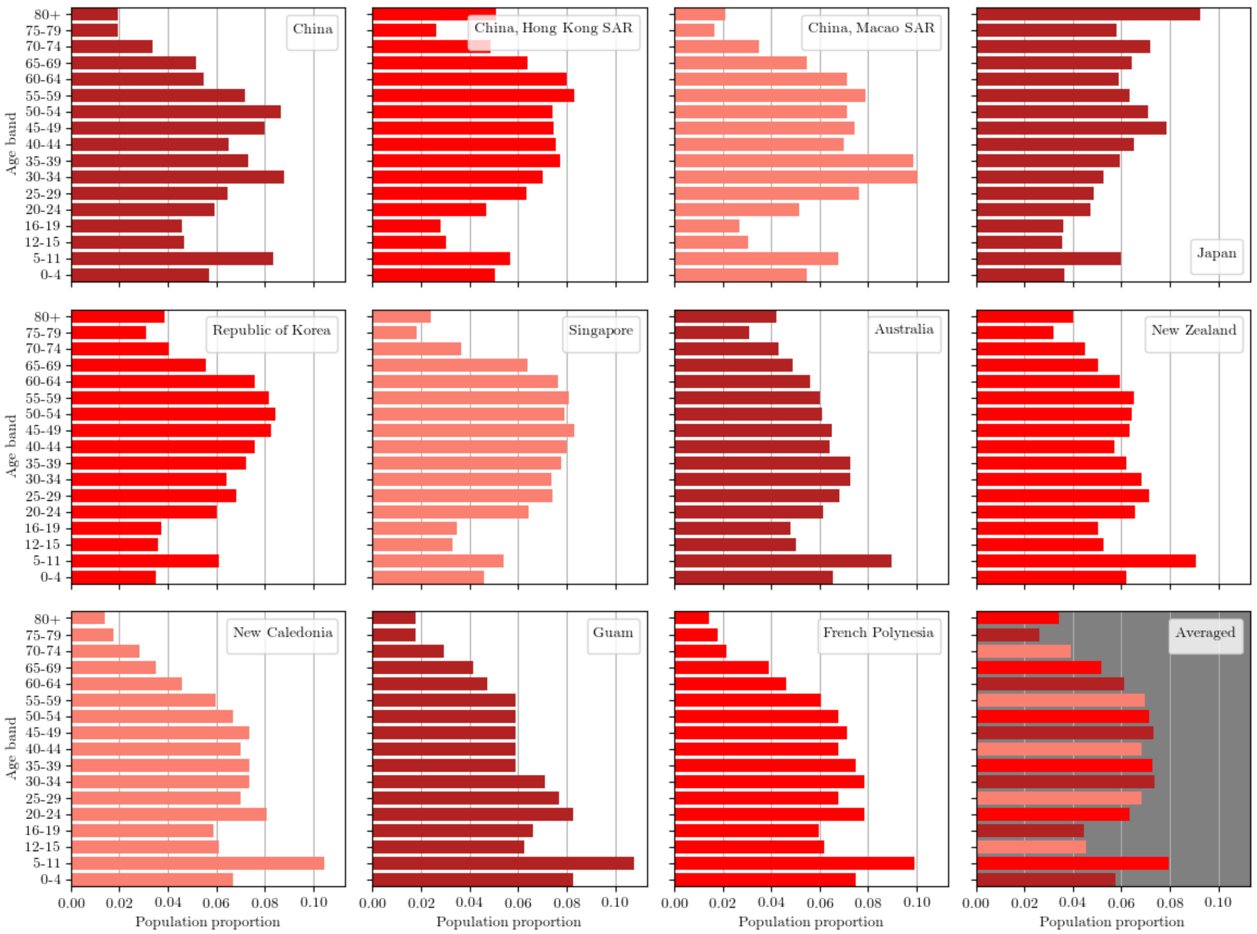

Figure A2: **Countries used to create the exemplar "older" population distribution**: relative proportions across age groups were averaged.

among the 5–64 age group.

2. In the second stage, the second dose is given to the same individuals as the first stage. The 65+ individuals receive their second doses first (though their exact dates are random). Once all the 65+ individuals that will be vaccinated are vaccinated, the 5–64 groups are vaccinated at random days. At the end of the second stage, $V\%$ of the population have received the primary course, where in the main paper, $V = 20, 50, 80$.

3. In the third stage, 80% of the fully vaccinated population receive a booster dose, while remaining available doses are given out as new primary course doses. During the third stage, 80% of daily doses are used as booster doses (for any age group 5+), while 20% of daily doses are used as new primary course doses (for any age group 5+).

Note that we impose a minimum 4 weeks between the first and second dose, and a minimum of 12 weeks between the second dose and the booster dose.

Suppose we want an initial $V\%$ vaccination coverage. We assume that the per-stage vaccination capacity

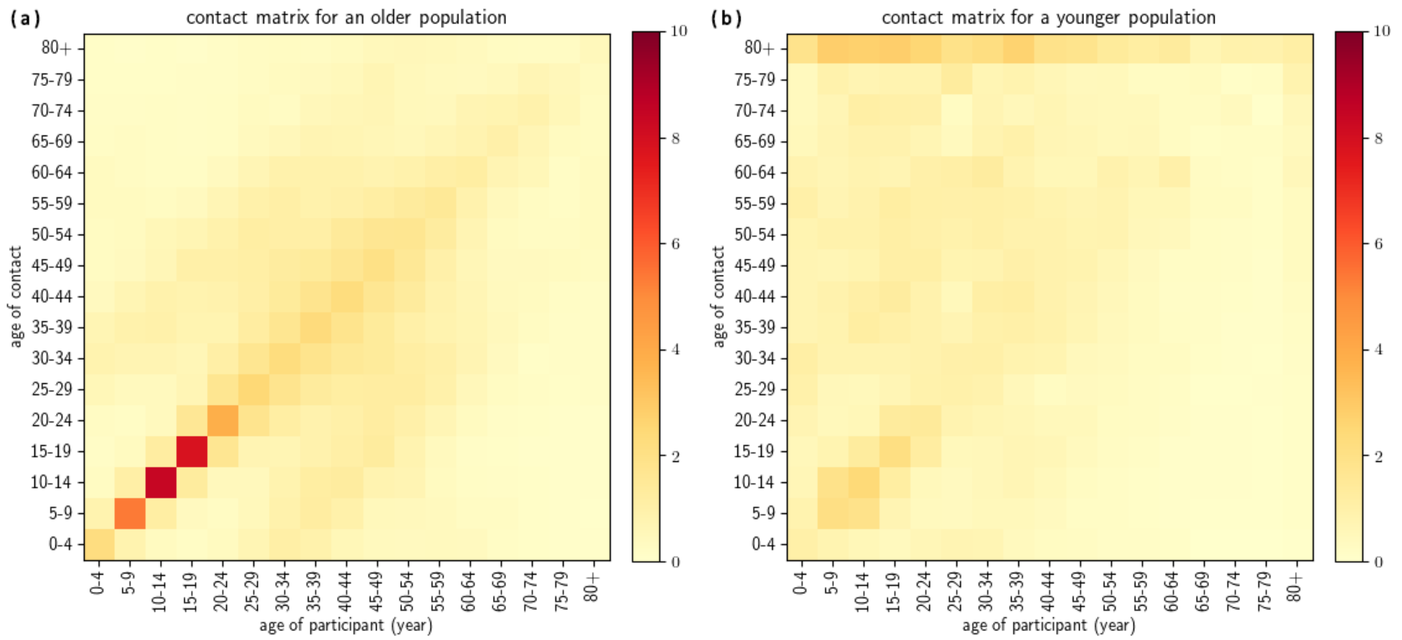

Figure A3: **Contact matrices used**, for the (a) older population, and (b) younger population.

is $VC = 100,000 \times V\%$ doses, that is, we will have VC doses per stage, or $VC/26$ doses per week. Thus, in the first stage, we have VC first doses. In the second stage, we have VC second doses. And in the third stage, we have $VC \times 80\%$ booster doses and $VC \times 20\%$ new primary course doses (which means $VC \times 10\%$ new first doses and $VC \times 10\%$ new second doses).

The vaccine allocation broadly follows WHO guidelines, which recommends prioritising the vaccination of older and higher-risk groups. At the lower vaccination rate of 20%, we ensure 80% of the 60+ age group are fully vaccinated by the first year. At the higher vaccination rates of 50% and 80%, we ensure that 95% of the 60+ age group are fully vaccinated by the end of the first year. Remaining doses available are distributed evenly among the lower age groups. An example of this allocation is given in Fig. A5.

A.3 Extended results

Fig. A6 shows the near-future ICU admissions for the various populations and scenarios. Overall, the trends in ICU admissions reflect the trends for deaths: that older populations typically have worse outcomes, and that vaccination can modestly reduce ICU admission numbers in all scenarios provided that vaccination coverage was 50% or above.

Fig. A7 demonstrates the range of outcomes of multiple simulations. Fig. A7(a) and (b) show infections averted by vaccination compared with scenarios representing the baseline no-vaccination case, while Fig. A7(c) and (d) show deaths averted by vaccination compared with scenarios representing the baseline no-vaccination case.

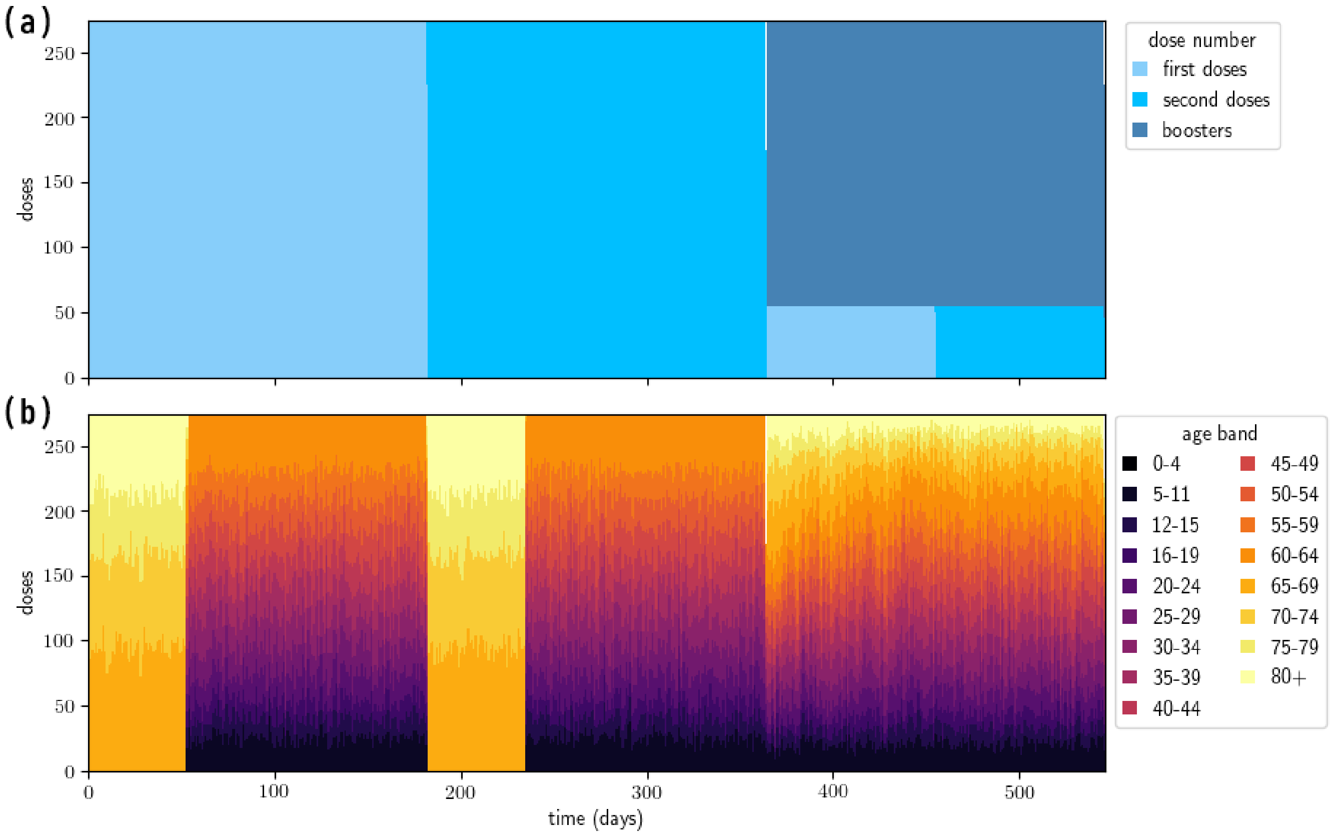

Figure A4: **Vaccine schedule and distribution example**: older population with 50% vaccination coverage after the first year. In the top panel, we see that doses are given out in the order of first doses, second doses, then jointly boosters and new primary doses. In the bottom panel, we see that during the first two stages, the older population 65+ are given their dose (numbered one or two) first, followed by the rest of the population. Meanwhile during the third (booster) stage, multiple different age groups are vaccinated daily, either with boosters or with new primary doses (with no age prioritisation for the booster dose).

At 80% vaccine coverage, the number of infections averted increases steadily in relation to the first wave attack rate which reflects the degree of transmission in the population. As vaccine coverage falls, the modelled maximum number of infections averted starts to decline for higher transmission pressures/attack rates.

Overall, in an older population, more deaths can potentially be prevented by vaccination than in a younger population. At 80% vaccine coverage, the number of deaths averted increases steadily in relation to the first wave attack rate which reflects the degree of transmission in the population. As vaccine coverage falls, the modelled maximum number of deaths averted starts to decline for higher transmission pressures/attack rates.

Fig. A8 shows the avoided infections and avoided deaths due to vaccination compared with scenarios representing the baseline no-vaccination case, in the scenarios where the second wave is due to an immune escape variant. We see that as vaccination coverage increases, we have increases in averted infections.

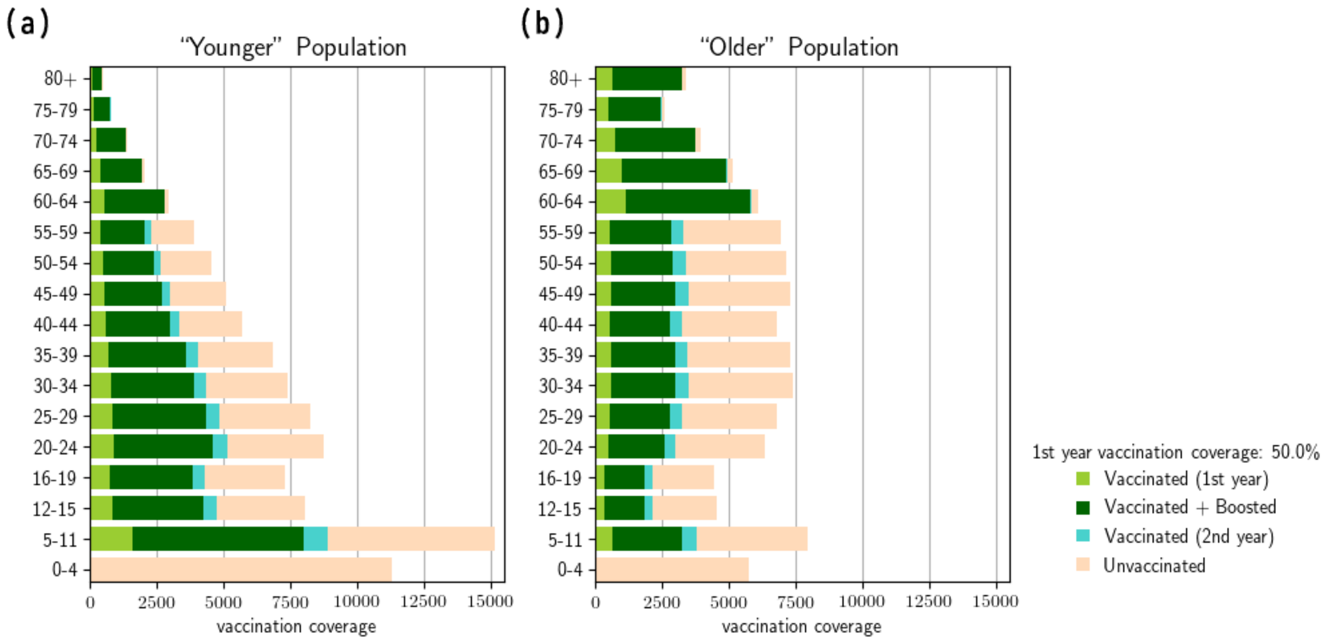

Figure A5: **Vaccine distribution example**: older and younger population with 50% vaccination coverage. The 65+ age group can be vaccinated to a maximum in-group coverage of 95%, while remaining available doses are distributed uniformly to the remaining 5–64 year old groups.

In Fig. A9, we considered different simulation end-times. If we end the simulation immediately after the vaccination ends at 1.5 years, i.e. at $t = 546$, we cannot see much difference between the different vaccination coverage settings—the full effects of vaccination have not played out yet. If we take an extra 30 days (compared to the main paper) and stop at $t = 680$, we see the overall same results as $t = 650$, showing that we did not need to include more days.

A.4 50% vs 80% booster coverage

In the main text, 80% of the initially vaccinated population were given boosters, while the remaining vaccination capacity was used to give new primary doses to previously unvaccinated individuals (note that all individuals vaccinated during the first year prior to boosters received the full primary course). In an alternative scenario, we could provide boosters to only 50% of the initially vaccinated population and try to vaccinate more people who had not yet been vaccinated. Fig. A10 shows the total difference in infection numbers and death numbers between the two scenarios, of which there is not a large noticeable difference.

B If vaccination efficacy was worse

Vaccine efficacy is important in determining just how much effect vaccination can have on future outcomes. In this section, we briefly consider a scenario where vaccine efficacy is lowered, with the

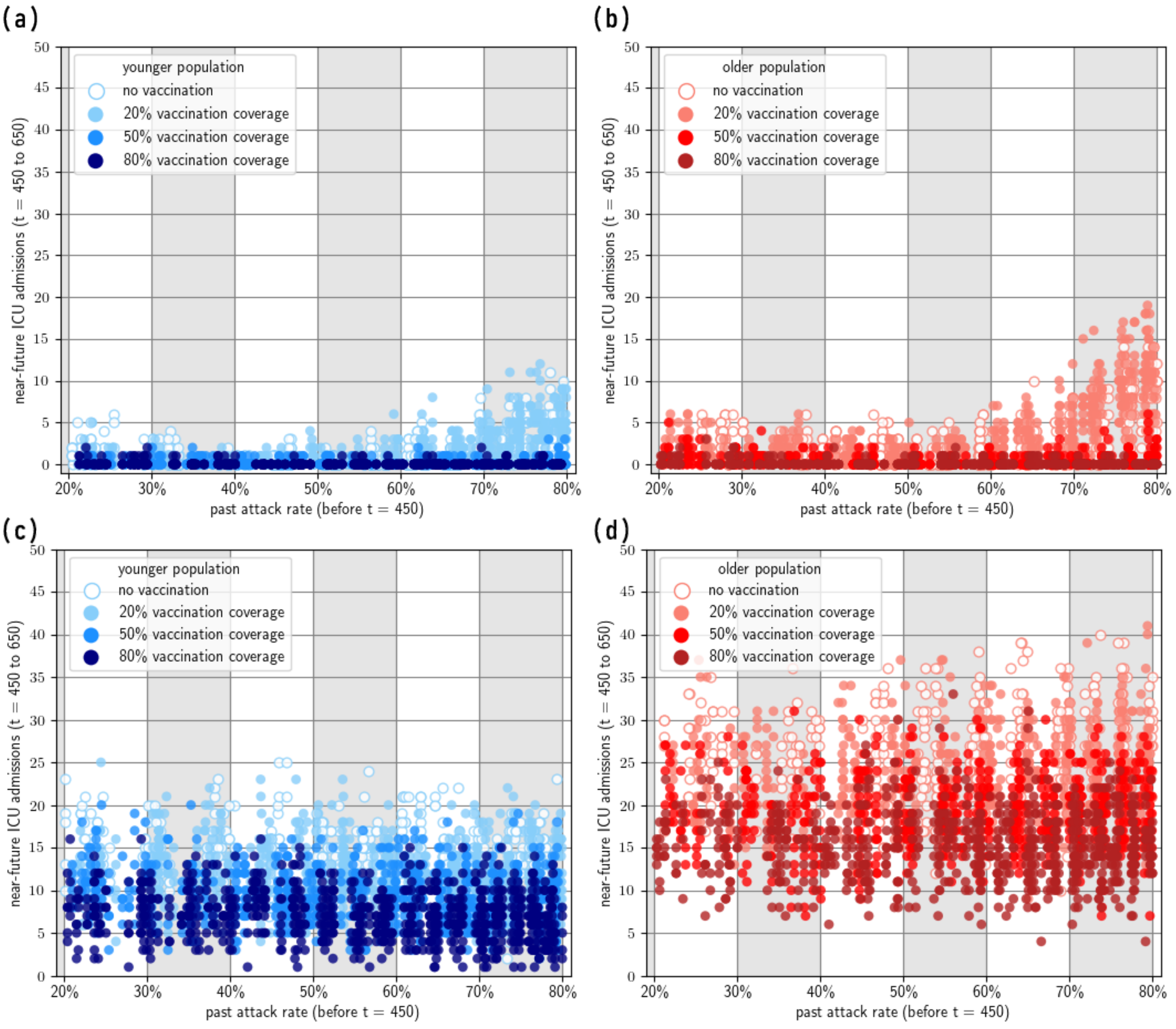

Figure A6: **Near-future ICU admissions** ($450 \leq t \leq 650$) given past attack rate ($t < 450$). Increased vaccination coverage reduces the upper limit of ICU admissions. Top row: second wave due to the same variant. Bottom row: second wave due to a BA4/5-like variant.

new parameters given in Table B1. The results are given in Fig. B1. With a worse vaccine, we find less difference in future infection/future attack rate given different vaccine coverages. Compared to the results in the main paper with a better vaccine, in the scenarios here, there are higher future attack rates and also higher numbers of severe outcomes.

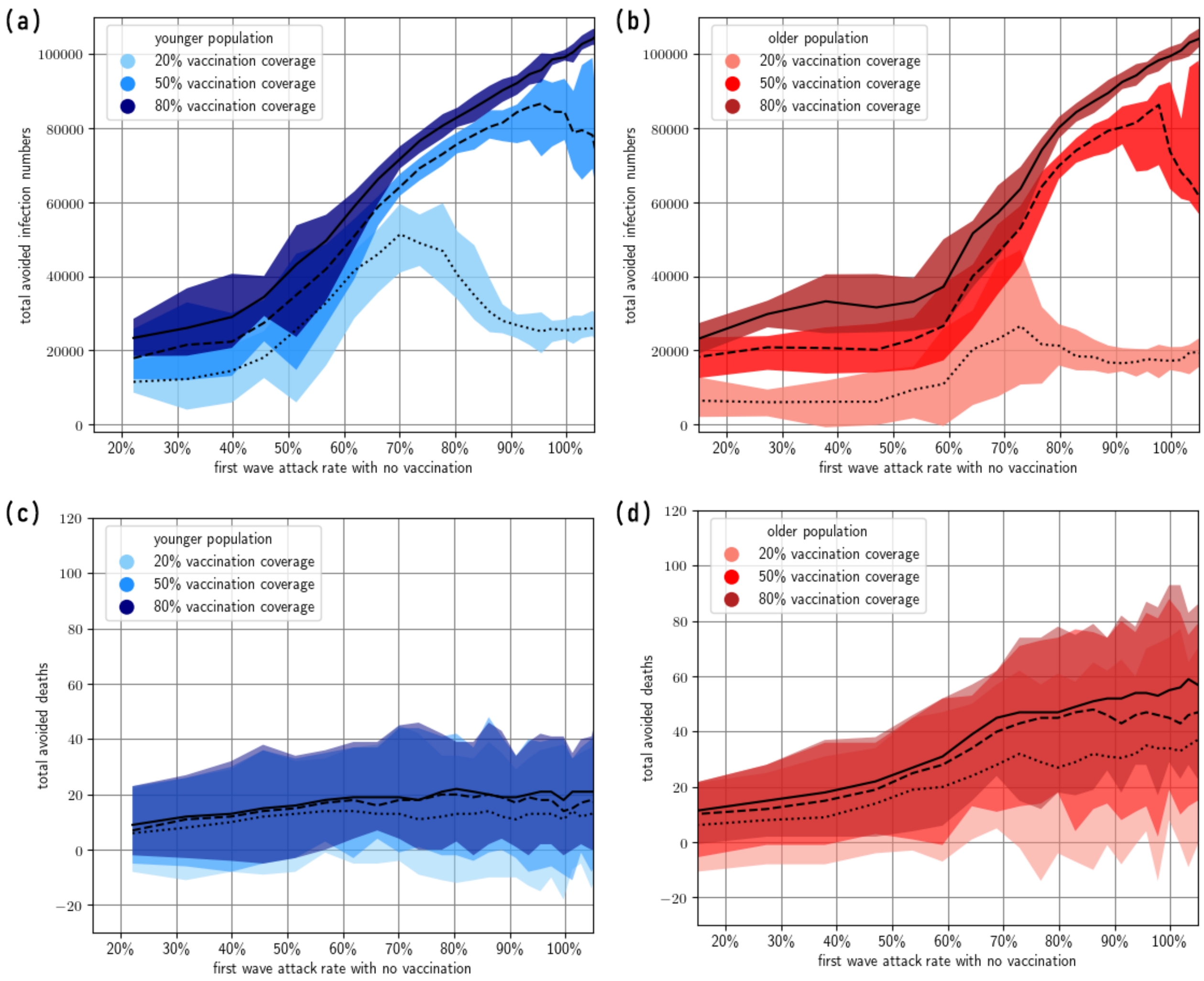

Figure A7: **Total avoided infections and total avoided deaths (where the second wave is due to the same variant)**. We compare the vaccination scenarios with the scenario with no vaccination throughout the full simulation. The horizontal axis is the past (first wave) attack rate of the scenario with no vaccination. Note that the upper limit in total avoided deaths is the same within each plot (c) and (d), as there exists some simulations where no deaths occur. The width of each colour band reflects the minimum/maximum of simulations exploring three levels of vaccine coverage (the lines show the median).

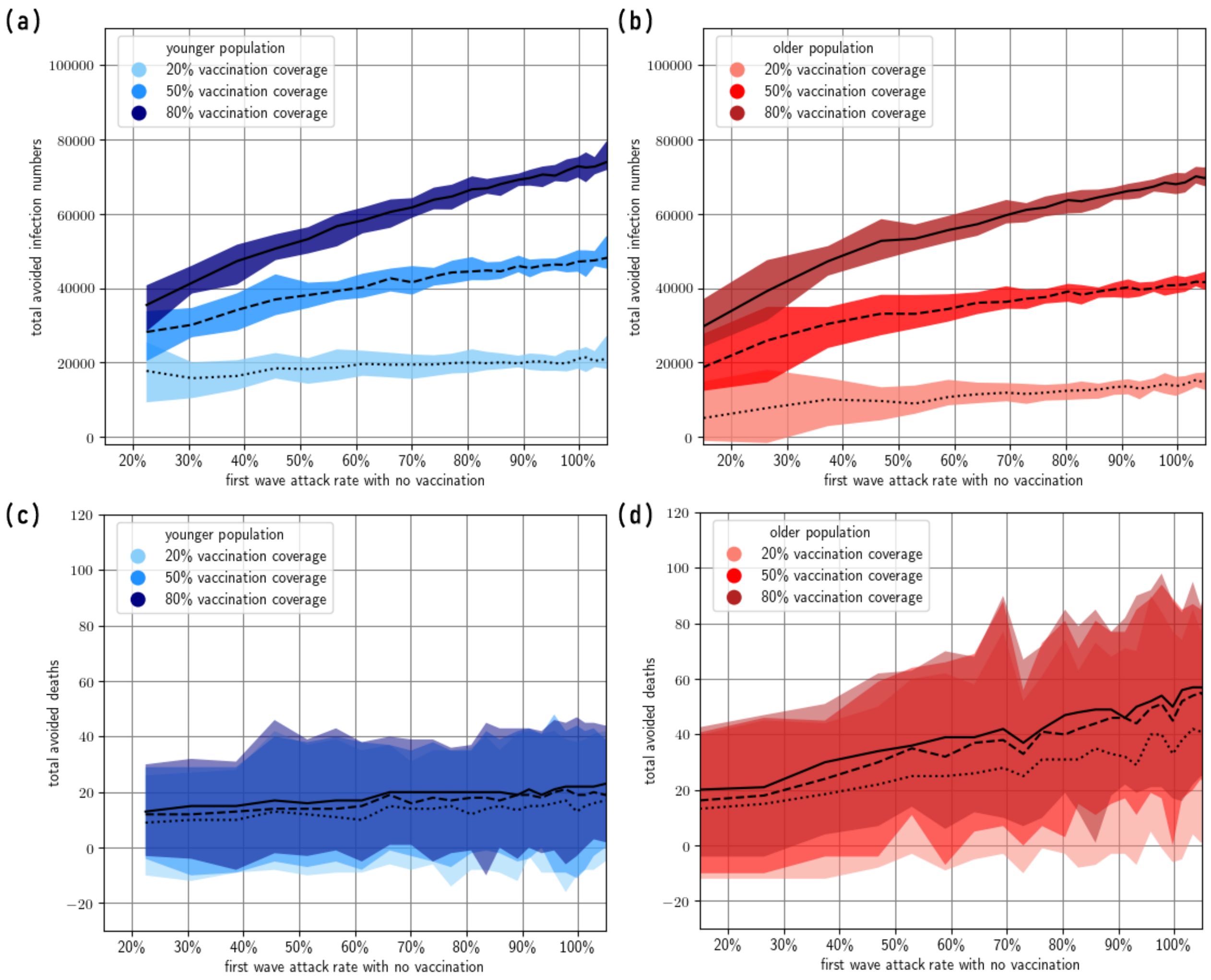

Figure A8: **Total avoided infections and total avoided deaths (where the second wave is due to a BA4/5-like immune escape variant)**. We compare the vaccination scenarios with the scenario with no vaccination throughout the full simulation. The horizontal axis is the past (first wave) attack rate of the scenario with no vaccination. The width of each colour band reflects the minimum/maximum of simulations exploring three levels of vaccine coverage (the black lines show the median).

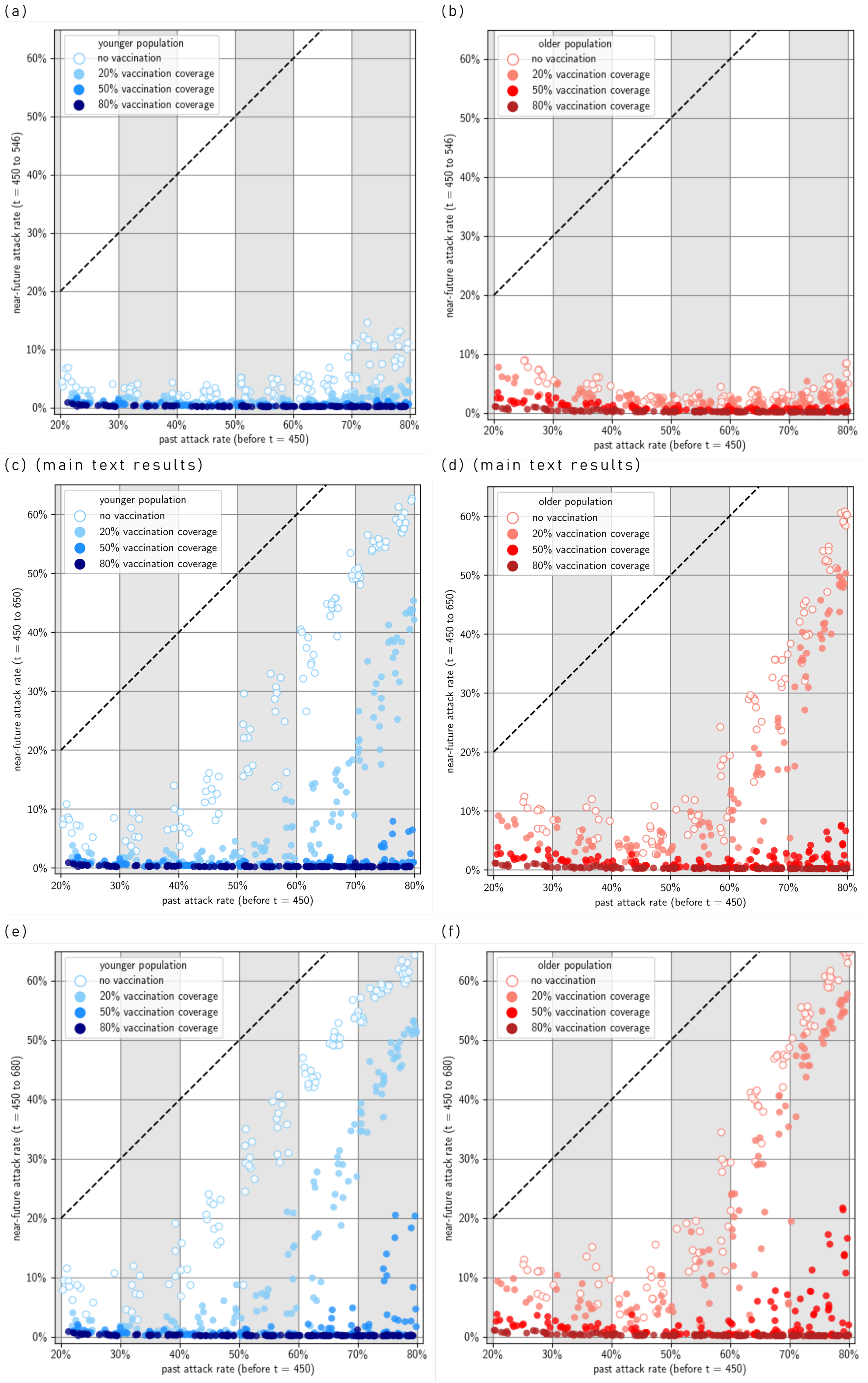

Figure A9: **Near-future attack rates given past immunity, for different "near-future" definitions.** (a, c, e) Younger population; (b, d, f) Older population. Top row: we consider a future of less than 100 days, ending when the third stage of vaccination ended ($t = 546$). Middle row: results from the main paper for reference (stopping at $t = 650$). Bottom row: including an extra month compared to the main paper results (stopping at $t = 680$).

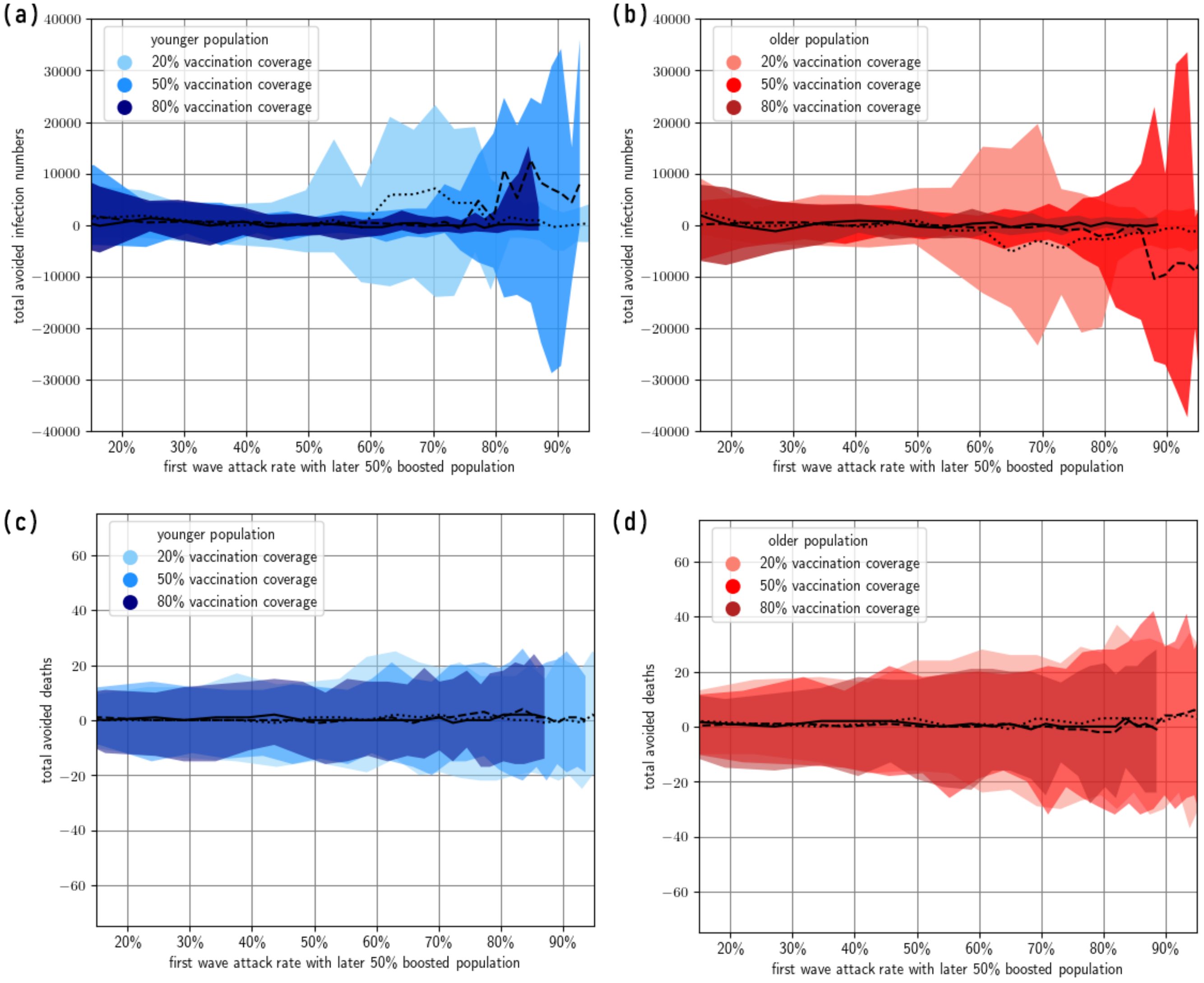

Figure A10: **Difference between infections and deaths for 50% vs 80% booster coverage allocation** (and where remaining doses are given out as new primary doses). Assumes that the second wave is due to the same variant.

Table B1: **Changed model parameters used for a lower efficacy vaccine.** Source: Ref. [4]

**Parameter: description**	**Value**
μ^0_{AZ1}: $\log_{10}$ of the mean neutralising antibody titre after the first dose of AstraZeneca	-1.318173846
μ^0_{AZ2}: $\log_{10}$ of the mean neutralising antibody titre after the second dose of AstraZeneca	-0.939262177
μ^0_{P3}: $\log_{10}$ of the mean neutralising antibody titre after the first booster dose of Pfizer	-0.336224683
$\mu_{\text{infection}}$: $\log_{10}$ of the mean neutralising antibody titre additionally gained after infection	1.158362492
c_h: midpoint of logistic function Eq. (5) of protection against hospitalisation	-1.206211545
c_d: midpoint of logistic function Eq. (5) of protection against death	-1.183605217
c_ξ: midpoint of logistic function Eq. (5) of protection against acquisition	-0.471657741
c_τ: midpoint of logistic function Eq. (5) of protection against transmission	0.018459279
c_q: midpoint of logistic function Eq. (5) of protection against symptomatic disease	-0.634901845
$\log(k)$: governs the steepness of the logistic curve relating antibodies to protection against disease outcome	1.707366579
k_a: decay rate of neutralising antibodies	0.008502135

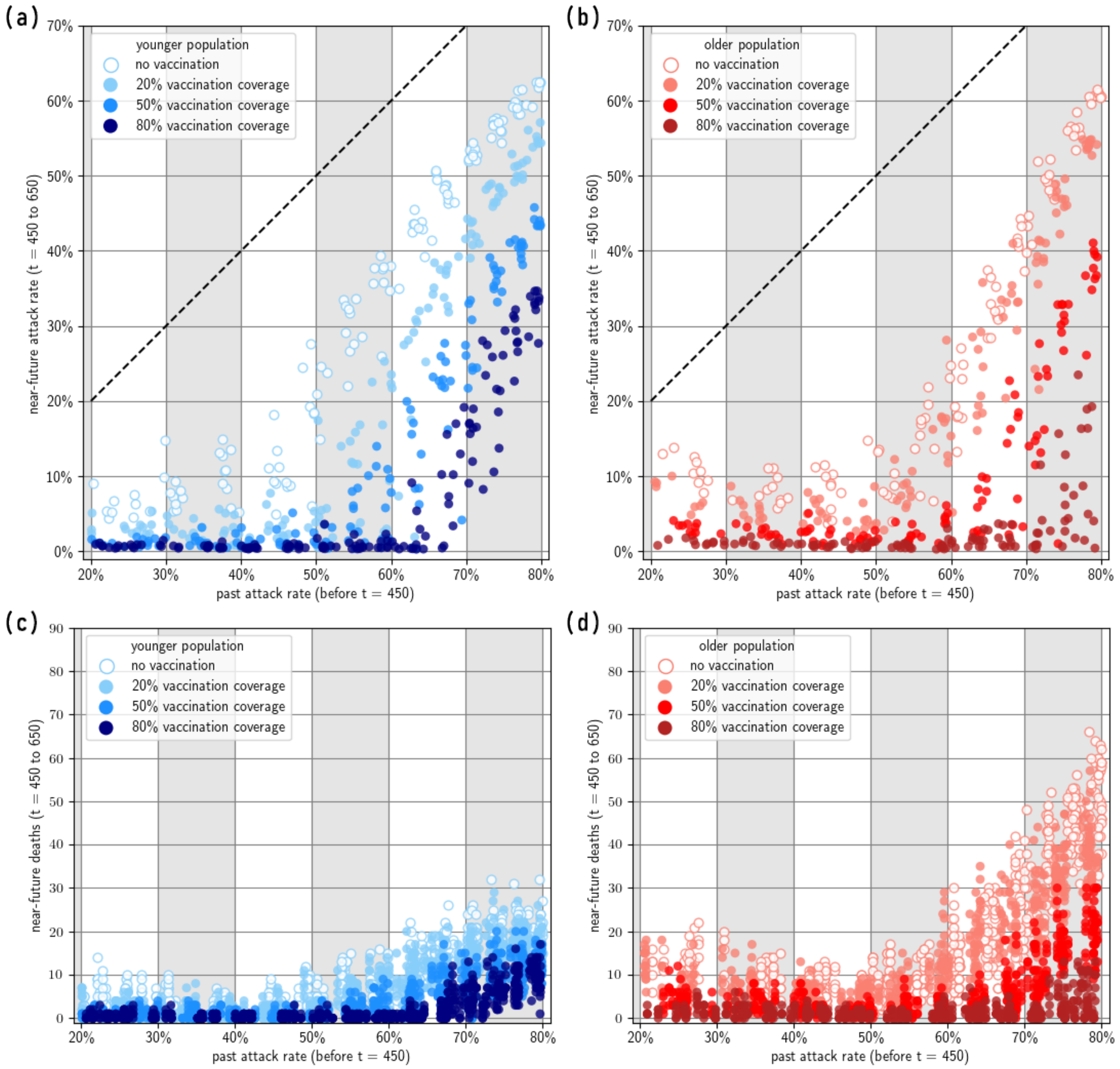

Figure B1: **Near-future attack rate and deaths given past immunity, for a worse vaccine.** Assuming a second wave due to the same Omicron BA.1 like variant.

Bibliography

[1] United Nations, Department of Economic and Social Affairs, Population Division. World Population Ageing 2019 Highlights, 2019. Accessed 13 May 2022. URL: https://www.un.org/en/development/desa/population/publications/pdf/ageing/WorldPopulationAgeing2019-Highlights.pdf.

[2] Sergei Scherbov and Warren Sanderson. New Measures of Population Ageing, 2019. Accessed 13 May 2022. URL: https://www.un.org/development/desa/pd/sites/www.un.org.development.desa.pd/files/unpd_egm_201902_s1_sergeischerbov.pdf.

[3] United Nations, Department of Economic and Social Affairs, Population Division. World Population Prospects 2019, custom data acquired via website., 2019. Accessed 13 May 2022. URL: https://population.un.org/wpp/DataQuery/.

[4] Nick Golding, Gerry Ryan, and Michael Lydeamore. Analyses to predict the efficacy and waning of vaccines and previous infection against transmission and clinical outcomes of SARS-CoV-2 variants., 2022. Github repository, Accessed 10 March 2023. URL: https://github.com/goldingn/neuts2efficacy.
